# Modulation of inducible nitric oxide synthase expression by sumoylation

**DOI:** 10.1186/1742-2094-6-12

**Published:** 2009-03-26

**Authors:** Candan A Akar, Douglas L Feinstein

**Affiliations:** 1 Department of Anesthesiology, University of Illinois, Chicago, IL 60612, USA; 2 Department of Veterans' Affairs, Jesse Brown VA, Chicago, IL 60612, USA

## Abstract

**Background:**

In astrocytes, the inflammatory induction of Nitric Oxide Synthase type 2 (NOS2) is inhibited by noradrenaline (NA) at the transcriptional level however its effects on specific transcription factors are not fully known. Recent studies show that the activity of several transcription factors including C/EBPβ, which is needed for maximal NOS2 expression, is modulated by conjugation of the small molecular weight protein SUMO. We examined whether the expression of SUMO Related Genes (SRGs: SUMO-1, the conjugating enzyme Ubc9, and the protease SENP1) are affected by inflammatory conditions or NA and whether SUMO-1 regulates NOS2 through interaction with C/EBPβ.

**Methods:**

Bacterial endotoxin lipopolysaccharide (LPS) was used to induce inflammatory responses including NOS2 expression in primary astrocytes. The mRNA levels of SRGs were determined by QPCR. A functional role for SUMOylation was evaluated by determining effects of over-expressing SRGs on NOS2 promoter and NFκB binding-element reporter constructs. Interactions of SUMO-1 and C/EBPβ with the NOS2 promoter were examined by chromatin immunoprecipitation assays. Interactions of SUMO-1 with C/EBPβ were examined by immunoprecipitation and Western blot analysis and by fluorescence resonance energy transfer (FRET) assays.

**Results:**

LPS decreased mRNA levels of SUMO-1, Ubc9 and SENP1 in primary astrocytes and a similar decrease occurred during normal aging in brain. NA attenuated the LPS-induced reductions and increased SUMO-1 above basal levels. Over-expression of SUMO-1, Ubc9, or SENP1 reduced the activation of a NOS2 promoter, whereas activation of a 4 × NFκB binding-element reporter was only reduced by SUMO-1. ChIP studies revealed interactions of SUMO-1 and C/EBPβ with C/EBP binding sites on the NOS2 promoter that were modulated by LPS and NA. SUMO-1 co-precipitated with C/EBPβ and a close proximity was confirmed by FRET analysis.

**Conclusion:**

Our results demonstrate that SUMOylation regulates NOS2 expression in astrocytes, and point to modification of C/EBPβ as a possible mechanism of action. Targeting the SUMOylation pathway may therefore offer a novel means to regulate inflammatory NOS2 expression in neurological conditions and diseases.

## Background

Nitric oxide generated by Nitric Oxide Synthase type 2 (NOS2, also termed iNOS) contributes to disease progression in a variety of neurological diseases and conditions. NOS2 is the calcium-independent isoform of the NOS family whose expression is generally restricted, but is highly and rapidly induced upon activation by a variety of stimuli, among them cytokines and bacterial endotoxin lipopolysaccharide (LPS). Several investigators have confirmed NOS2 expression in primary astrocytes upon inflammatory induction [1-4] as well as in neurological diseases including Multiple Sclerosis (MS), its animal model EAE [5-7], cerebral ischemia [8], and Alzheimer's disease [9-11].

Transcription of the NOS2 gene has been shown to require activation of transcription factor NFκB [12,13]; however in many cell types activation of CCAAT/enhancer-binding proteins (C/EBPs) are necessary for maximal NOS2 expression [14-18]. The C/EBPs constitute a family of basic leucine-zipper transcription factors which form homo and hetero dimers that bind to similar cis-regulatory elements with varying affinities [19,20]. The leucine-zipper motif constitutes the dimerization domain of the C/EBP protein whereas the basic region is the DNA contact area that determines binding specificity. C/EBP isoforms include C/EBPα, C/EBPβ (also known as NF-IL6, IL-6-DBP, LAP, AGP/EBP, or CRP2), C/EBPδ (also called CRP3, CELF, or NF-IL6b), C/EBPγ (IgEBP), C/EBPε (CRP1), and C/EBPζ (CHOP). C/EBPβ is itself composed of several variants due to use of alternative translation start sites, resulting in C/EBPβ1 (the full length protein, also called LAP*), C/EBPβ2 (LAP, shorter by 23 amino acids versus LAP*), and C/EBPβ-20 (LIP, which lacks the transactivation domain and is considered to be a dominant negative isoform).

The expression of NOS2 has been shown to be regulated by the neurotransmitter noradrenaline (NA), via activation of beta-adrenergic receptors and a resulting increase in cAMP levels [21-23]. Although the effect of NA occurs at the transcriptional level, it does not inhibit the activation of NFκB or its binding to DNA [24]. A 27 bp region of the NOS2 promoter containing an almost consensus (6/7 identities) CRE site and a consensus binding site for C/EBP was shown to be critical for inhibition by NA raising the possibility of regulation through these transcription factors [25].

The activity of C/EBP family members, like many transcription factors and co-factors, is regulated by the protein Small Ubiquitin like Modifier (SUMO) [26-29]. The covalent binding of SUMO to acceptor sites on target proteins involves several steps starting with conversion of the precursor to mature SUMO by a C-terminal hydrolase (SENP) [30]. The SUMO Activating Enzyme (SAE) E1 transfers SUMO to conjugating enzyme E2 (Ubc9) which then catalyzes the formation of an isopeptide bond between the C-terminal glycine of SUMO and the ε-amino group of a target lysine. The majority of target lysines are within SUMO consensus motifs, however proteins without such motifs can also be SUMOylated. SUMO E3 ligases (PIAS, RanBP2) increase the efficiency of the reaction possibly also facilitating SUMOylation of targets lacking the consensus motif. The proteases (SENP) involved in maturation are also responsible for the removal of SUMO from substrates [31-33].

Recent reports implicating SUMOylation in regulation of inflammation [34-36] as well as NOS2 transcription [37] combined with our findings that effects of NA on NOS2 expression most likely involve C/EBPβ prompted us to investigate whether SUMO-1 is involved in regulation of NOS2 transcription through modification of C/EBPβ. In this study we show that the expression of SUMO-1 and two critical SUMOylation enzymes (collectively referred to here as SRGs for SUMO Related Genes) as well as C/EBPβ are regulated under pro- and anti-inflammatory conditions, and that these genes modulate NOS2 promoter activation. Our data further support that interactions of SUMO-1 and C/EBPβ occur at the NOS2 promoter and are important for modulating NOS2 transcription.

## Materials and methods

### Cells and treatments

Primary astrocytes were prepared from cerebral cortices of postnatal day 1 Sprague-Dawley rats as previously described [1]. After 2 weeks of growth in complete medium (DMEM, 10% FBS, 1% penicillin/streptomycin) the cultures consisted of 95–98% astrocytes. C6 rat glioma cells were grown in complete medium. Cell cultures at 80–90% confluency, were transferred to DMEM with 1% FBS and antibiotics and treated with LPS (1 μg/ml) and/or NA (30 μM) for induction or modulation of NOS2. Luciferase activity assays, chromatin and protein immunoprecipitations, and mRNA analysis were conducted 4 hr after treatments. Transfections were done with cells at 50–60% confluency and treatments were initiated 40 hr after transfection.

### mRNA analysis

**Figure 1:**
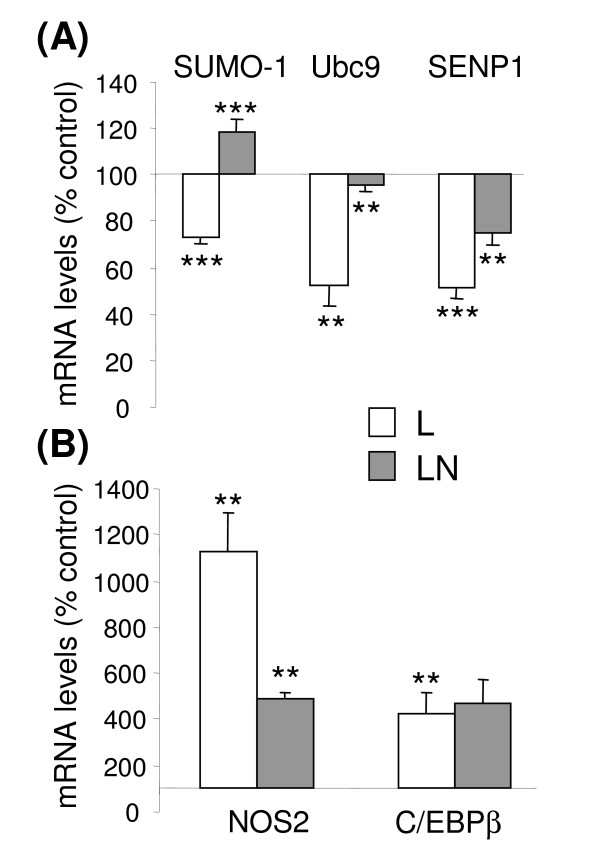
**Effect of inflammatory activation on SRGs, NOS2, and C/EBPβ mRNA levels in astrocytes**. Primary rat astrocytes were treated with LPS (L) or LPS+NA (LN) for 4 hours. Total mRNA was converted to cDNA, and amplified using primers for (**A**) SUMO-1, Ubc9, and SENP1; and (**B**) NOS2 and C/EBPβ. PCR products were separated through 2% agarose gels, and the band intensities were quantified using ImageJ software. The data is the mRNA level measured in treated samples compared to non-treated control samples, and normalized to values for β-actin measured in the same samples. Data is the mean ± sd of n = 3 samples in each group. **, p < 0.01; ***, p < 0.001 L vs control or LN vs L (unpaired t-test).

**Figure 2:**
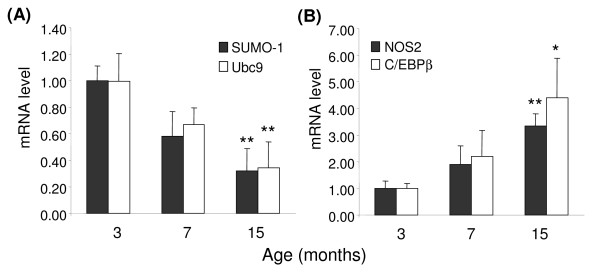
**Changes in SRG, NOS2, and C/EBPβ mRNA levels during aging**. Cerebral cortices of C57BL/6 mice of the indicated ages were sonicated briefly in Trizol reagent, total mRNA extracted, and converted to cDNA. Aliquots were analyzed by QPCR for levels of (**A**) SUMO-1 and Ubc9; and (**B**) NOS2 and C/EBPβ mRNAs. The data are mean ± sd of 3 mice per age group, normalized to values for β-actin mRNA measured in the same samples. There was a significant decrease in SUMO-1 and Ubc9, and a significant increase in NOS2 and C/EBPβ over time (P < 0.01, 1-way ANOVA); *, p < 0.05; **, p < 0.01 vs 3-month samples (Bonferroni multiple comparison post hoc tests).

Total cytoplasmic RNA was prepared from primary astrocytes or cerebral cortices of mice using TRIZOL reagent (Invitrogen, Carlsbad, CA); aliquots were converted to cDNA using random hexamer primers and mRNA levels estimated by reverse transcription-PCR (RT-PCR). The primers used for NOS2 were forward 5'-GGAGAAGGGGACGAACTCAGT-3' and reverse 5'-GCATTGGAAGTGAAGCGTTTC-3'; for SUMO-1 were forward 5'-TTTCATGTGTGCACAGAGAGGCCA-3' and reverse 5'-AGTCCAGGAGTGAAGCAACCACAT-3'; for Ubc9 were forward 5'-AAGGAGGCTTGTTCAAGCTACGGA-3' and reverse TTTGATGGTGATAGCTGGCCTCCA-3'; for SENP-1 were forward 5'-ACACTGGAGCCTGGTGGTAATTGA-3' and reverse 5'-TGTACTGCTTCCACTCCAAAGGGT-3'; for C/EBPβ were forward 5'-ATGCAATCCGGATCAAACGTGGCT-3' and reverse 5'-TTTAAGTGATTACTCAGGGCCCGGCT-3'; for β-actin were forward 5'-CCTGAAGTACCCCATTGAACA-3' and reverse 5'-CACACGCAGCTCATTGTAGAA-3'. PCR conditions were 35 cycles of denaturation at 94°C for 10 sec; annealing at 61°C for 15 sec; and extension at 72°C for 20 sec; followed by 2 min at 72°C. For the data shown in figure 1, changes in mRNA levels were estimated by semi-quantitative means. The PCR products were separated through agarose gels, product band intensities for SUMO-1, Ubc9, NOS2, C/EBPβ, and β-actin quantified by ImageJ software, and the mRNA levels for target genes normalized to the values measured for β-actin in the same samples. For the data shown in figure 2, changes in mRNA levels were estimated by quantitative PCR (QPCR) in the presence of SYBR Green (1:10,000 dilution of stock solution; Molecular Probes, Invitrogen) carried out in a 20-μl reaction in a Corbett Rotor-Gene (Corbett Research, QIAGEN, Valencia CA). Relative mRNA levels were calculated from the take-off cycle (Ct) of reactions using manufacturer's included software, and normalized to values measured for β-actin mRNA in the same samples to account for any differences in sample quality or tube to tube variability. QPCR measurements did not show any significant change in the Ct values for β-actin, nor for those measured for α-tubulin, in brain cortical RNA samples from mice aged 3 to 15 months (data not shown). QPCR products were separated by electrophoresis through 2% agarose gels containing 0.1 μg/ml ethidium bromide to verify production of correctly sized products.

### Plasmids

Luciferase reporter plasmids were constructed with NOS2 promoter fragments as described [25]. The pGL3-2.2 plasmid contains 2.2 kB upstream of the start site of the rat NOS2 promoter; pGL3-CREB extends upstream to base -187 relative to the start site; and pGL3-κB extends upstream to base -130. HA-SUMO-1, and myc-Ubc9 expression plasmids were a gift of Dr. Edward Yeh (Anderson Cancer Center, Houston, TX) and pMSV-C/EBPβ a gift of Dr. Steven J. Ackerman (University of Illinois, Chicago, IL). pSV(X)-C/EBPβ was obtained from Addgene (Cambridge, MA; plasmid 13393; donated by Dr. Richard Schwartz). YFP-SUMO-1 and CFP-RanGAP1 were a gift of Dr Frauke Melchior (Georg-August-Universitaet, Goettingen, Germany). To construct CFP-C/EBPβ, RanGAP1 was excised using Bgl2 and BamH1 from the CFP-plasmid and replaced with the C/EBPβ insert excised using BamH1 from pSV(X)-C/EBPβ.

### Transfections

Stable transfectants of NOS2 constructs in pGL3 vector were generated as previously described [25]. Transient transfections were conducted using Effectene reagent (QIAGEN) according to manufacturers instructions. The phRG-TK vector (Promega, Madison, WI) was co-transfected as an internal control reporter to experimental reporter constructs at a ratio of 1:50 (w/w).

### Luciferase activity assays

Luciferase activity in stably transfected cells was determined using Bright-Glo Luciferase Assay System (Promega) in a microplate luminometer (Rosys-Anthos, Anthos, Durham, NC, USA). Luciferase activity in transiently transfected cells was determined using Dual-Luciferase Reporter Assay System (Promega) in a single sample luminometer (Femtomaster FB12, Zylux, Huntsville, AL).

### Chromatin immunoprecipitation assays

Chromatin immunoprecipitation (ChIP) experiments were conducted according to a protocol developed at the University Health Network Microarray Centre , Toronto, Canada). Briefly, cells grown in 10 cm plates to 80–90% confluency were treated as described above, proteins were cross linked to DNA using 1% formaldehyde (37°C for 10 min), then cells were lysed in buffer containing 1% SDS, 10 mM EDTA and 50 mM Tris, pH 8. Chromatin was sheared by sonication on ice at setting 6 (Sonic Dismembrator, Fisher Scientific, Pittsburgh, PA) for 20 s, 8× with 2 min intervals. Supernatants containing chromatin fragments (mostly 600 bp) were incubated with 0.5 μg/ml mouse monoclonal IgG2a (sc-7962 directed against C-terminus amino acids 199–345 of human C/EBPβ and which detects all C/EBPβ isoforms and to a lesser extent C/EBPα, δ, or ε, Santa Cruz Biotechnology, Santa Cruz, CA); 0.5 μg/ml mouse monoclonal IgG (anti-GMP1, which recognizes the 17 kDa unconjugated SUMO-1 as well as SUMO-1 ligated to target proteins, Zymed, Invitrogen) or no antibody (control) overnight. Immunocomplexes were precipitated with Protein A Sepharose beads in the presence of 1 mg/ml yeast tRNA as blocking agent. Precipitates were subjected to several washes and then protein-DNA complexes eluted from beads by incubation in 0.1 M NaHCO_3 _containing 1% SDS. DNA: protein cross-links were reversed by incubating samples at 65°C for 5 hr in the presence of 200 mM NaCl and 20 μg/ml RNAse A, proteins digested with proteinase K (0.3 mg/ml), and DNA recovered using QiaQuick (QIAGEN) PCR purification columns. C/EBPβ binding site containing segments of the rat NOS2 promoter were amplified using primers -425F 5'-TCCACACTGCCAGTAATCCACAGA-3' and -107R 5'-CCAGTAGGGTGTGCAAGTTAG-3' and primers -127F 5'-GCTAACTTGCACACCCTACTG-3' and 61R 5'-TGGCTGAGAAGTTTCAAACCAGCG-3' For sequential ChIP experiments precipitated chromatin was eluted from the protein A/G-sepharose beads (Santa Cruz) with 10 mM DTT, diluted 20× with 20 mM Tris (pH 8) containing 150 mM NaCl, 2 mM EDTA, 1% TritonX-100 and subjected to a second round of immunoprecipitation.

### Immunoprecipitation and western blot analysis

Astrocytes grown in 6 cm plates were treated as described above. Cells were lysed in β-glycerophosphate buffer containing 2% SDS as described [38]. Immunoprecipitations were conducted with 1 μg/ml mouse monoclonal IgG2a (sc-7962, Santa Cruz); after lysates were diluted 20× with lysis buffer without SDS. Precipitated proteins were separated by 10% SDS-PAGE and transferred onto PVDF membranes. Membranes were blocked with 5% milk in TBS-T and probed with rabbit polyclonal anti-SUMO-1 antibody (0.87 μg/ml final concentration) raised against mature human SUMO-1 [39], a gift of Dr. John M. Hallenbeck, NINDS/NIH, Bethesda, MD).

### Fluorescence Resonance Energy Transfer analysis (FRET)

Primary astrocytes on gelatin coated coverslips were co-transfected with YFP-SUMO-1 and CFP-C/EBPβ expression plasmids. 24 hr after transfection cells were fixed with 3.7% formaldehyde. FRET analysis was conducted using LSM510 Meta system (Zeiss, Thornwood, NY). Emission spectra were obtained from cells co-expressing YFP-SUMO-1 and CFP-C/EBPβ as three dimensional image stacks by laser scanning microscopy using non bleaching intensities of 458 nm for CFP and 514 nm for YFP. Reference spectra obtained from cells expressing only CFP-C/EBPβ or only YFP-SUMO-1 were used for separation of the emission spectra by linear un-mixing software of the LSM510 Meta system. In cells expressing both fluorophores the acceptor YFP-SUMO-1 was photobleached in defined regions of interest (ROIs) using 100% power of the 514 nm laser line and the lack of fluorescence resonance energy transfer to the acceptor in those regions was observed as an increase in the donor (CFP-C/EBPβ) fluorescence.

### Statistical analysis

**Figure 3:**
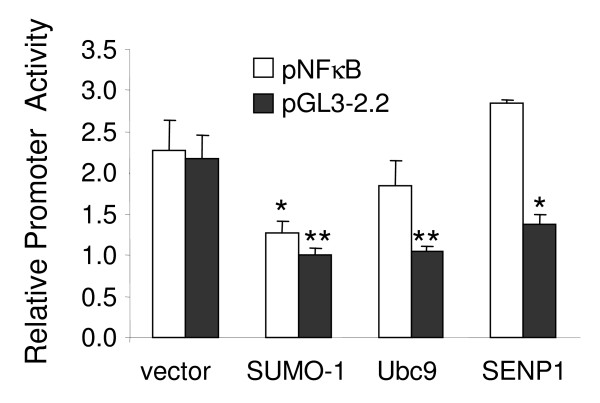
**Effect of increasing SRG expression on promoter activity**. C6 cells stably transfected with pNFκB (4 × NFκB binding-element) or pGL3-2.2 (2.2 kB NOS2 promoter sequence) luciferase reporter plasmids were transiently transfected with SUMO-1, Ubc9, or SENP1 expression plasmids or with vector only (pcDNA3). After 40 hours cells were treated with LPS for 4 hours and luciferase activity measured. The data shows relative luciferase activity of treated versus untreated cells, and is the mean ± sd of 3 independent experiments. *, p < 0.05; **, p < 0.01 vs vector (unpaired t-test).

**Figure 4:**
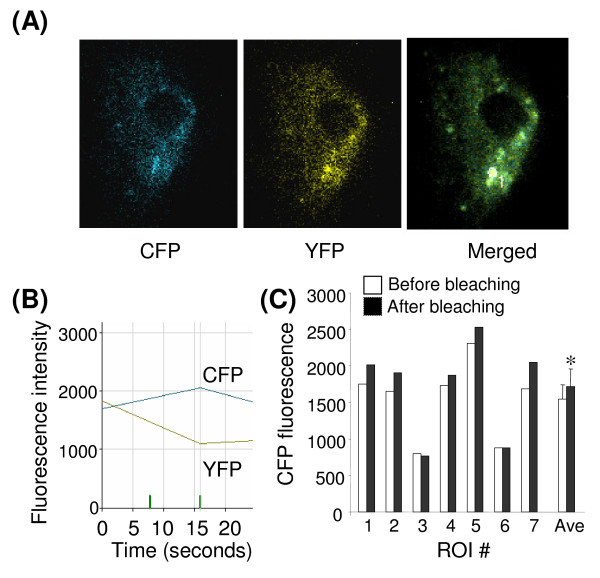
**Sumoylation of C/EBPβ in astrocytes**. Primary rat astrocytes were co-transfected with YFP-SUMO-1 and CFP-C/EBPβ expression plasmids. After 24 hr the cells were fixed, and cells co-expressing YFP-SUMO-1 and CFP-C/EBPβ were subjected to FRET analysis. (A) Representative image of CFP and YFP fluorescence after linear un-mixing of emission spectra and merged image showing the region of interest (ROI) analyzed. (B) FRET data after acceptor photobleaching of the ROI indicated ("1") in panel A, showing an increase in CFP fluorescence coincident with a decrease in YFP fluorescence. (**C**) CFP fluorescence of ROIs measured in 7 different cells before (open bars) and after (filled bars) acceptor photobleaching. "Ave" mean ± s.e (n = 7) of CFP fluorescence. *, p < 0.05 after versus before, non parametric paired T-test.

Group data shown in figures 1 and 3 were analyzed by unpaired T-tests; QPCR data at different ages shown in figure 2 was analyzed by 1 way ANOVA and Bonferroni multiple comparison post hoc analyses; FRET data shown in figure 4 was analyzed by non-parametric paired T-test of CFP fluorescence measured before and after photobleaching in the same ROI. Significance was taken at p values < 0.05.

## Results

### SRG expression is modulated by inflammatory stimulus and NA

The mRNA levels of the SRGs SUMO-1, Ubc9, and SENP1 were determined under naïve or inflammatory conditions in primary astrocytes (Figure 1). Treatment with LPS resulted in an approximately 50% decrease in Ubc9 and SENP1 mRNA levels, and an approximately 30% decrease in SUMO-1 mRNA levels compared to untreated (control) cells. The presence of NA altered the LPS effect on these enzymes and SUMO-1 to varying degrees. The inhibitory effect of LPS on SENP1 was attenuated by approximately 50%, whereas the level of Ubc9 mRNA was restored back to untreated levels. NA not only reversed the LPS effect on SUMO-1 mRNA levels but caused an increase of approximately 20% (Figure 1A). The inflammatory effect of LPS was verified by an increase in NOS2 mRNA (Figure 1B). C/EBPβ mRNA levels were also increased by LPS, consistent with its involvement in inflammatory activation. As previously shown [23], co-treatment with NA reduced the effect of LPS on NOS2 but had no effect on C/EBPβ mRNA levels (Figure 1B).

### SRG expression decreases with aging

During aging, a reduction in brain NA levels occurs, which could contribute to increased neurological damage [40]. Analysis of expression of SUMO-1 and the conjugating enzyme Ubc9 mRNA levels (Figure 2A) revealed a significant age-dependent change in SUMO-1 and Ubc9 mRNA levels in the frontal cortex of wild type C57BL/6 mice between 3 and 15 months of age, with values at 15 months reduced about 70% compared to the values measured at 3 months. During this time, there was also a significant age-dependent change in C/EBPβ and NOS2 mRNA levels, with increases of 3- to 5-fold measured between 3 and 15 months of age (Figure 2B) suggesting an age-dependent increase in overall inflammatory status.

### Over-expression of SRGs modulate NOS2 promoter activity

To determine if SUMOylation was involved in inflammatory activation, rat C6 glioma cells stably transfected with luciferase reporter constructs pNFκB (4 × NFκB binding-element) or pGL3-2.2 (containing the 2.2 κB NOS2 promoter sequence)were transiently transfected with SRG expression plasmids and the promoter activation by LPS was determined (Figure 3). Over-expression of SUMO-1 diminished the LPS-dependent increase in activity of both constructs. Increasing Ubc9 levels did not affect pNFκB activity but decreased pGL3-2.2 activity. Similarly, over-expressing SUMO protease SENP1 did not affect pNFκB but diminished pGL3-2.2 activity.

### Effects of LPS and NA on association of C/EBPβ and SUMO-1 with the NOS2 promoter

**Figure 5:**
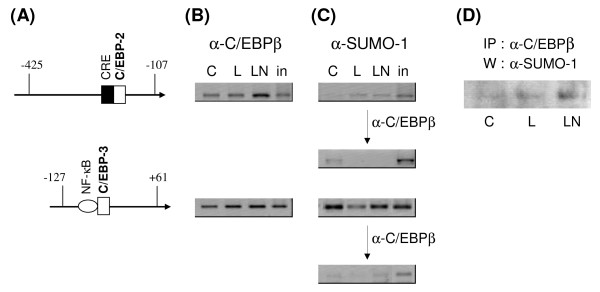
**Presence of C/EBPβ and SUMO-1 on the NOS2 promoter**. Primary astrocytes were treated with nothing (C), LPS (L), or LPS+NA (LN) for 4 hours, then ChIP analysis conducted. (**A**) Two fragments of the rat NOS2 promoter containing the proximal C/EBP-2 (top) or C/EBP-3 (bottom) binding sites were amplified by PCR after immunoprecipitation using (**B**) anti-C/EBPβ antibody or (**C**) anti-SUMO-1 antibody alone or followed by anti-C/EBPβ antibody. The products were separated through 2% agarose gels and their accuracy was verified by comparison to input DNA ("in", 5% of total input). The images shown are inverted versions (Adobe Photoshop) of the original gel images and are representative of results obtained in 2 separate experiments. (**D**) Lysates of astrocytes treated as indicated above were immunoprecipitated (IP) with anti-C/EBPβ antibody and Western blot (W) of the samples was probed with anti-SUMO-1 antibody.

The involvement of C/EBPβ and SUMO-1 in NOS2 transcription was further investigated using ChIP assays to detect the association of these proteins with regions of the NOS2 promoter containing either the C/EBP-2 or the C/EBP-3 site (Figure 5A). C/EBPβ was found associated with both the C/EBP-2 and C/EBP-3 sites under all conditions; however binding to C/EBP-2 was increased by treatment with LPS plus NA (Figure 5B). SUMO-1 was detected at both sites, although a stronger signal was obtained with the fragment of the NOS2 promoter containing the C/EBP-3 site (Figure 5C). Treatment with LPS reduced association of SUMO-1 with this site while addition of NA restored it (or prevented that loss). Re-precipitation of SUMO-1 interacting fragments with anti-C/EBPβ antibody showed that under all conditions, the C/EBP-3 site also interacted with C/EBPβ (Figure 5C, **bottom panel**). In contrast there was little evidence for simultaneous association of both SUMO-1 and C/EBPβ at C/EBP-2 site. An interaction of SUMO-1 with C/EBPβ was confirmed by Western blots which showed that SUMO-1 co-immunoprecipitated with C/EBPβ (Figure 5D), and this interaction was increased by the presence of NA.

### Interaction of SUMO-1 and C/EBPβ

The possibility that C/EBPβ can be SUMOylated in primary astrocytes was further tested using FRET analysis. Primary astrocytes were co-transfected with expression plasmids encoding CFP-tagged C/EBPβ and YFP-tagged SUMO-1 (Figure 4A), and FRET measured by the increase in donor fluorescence (CFP-C/EBPβ) after photobleaching of the acceptor molecule (YFP-SUMO-1) (Figure 4B). Acceptor photobleaching of selective ROIs in cells expressing both fluorophores resulted in a significant increase of donor fluorescence (Figure 4C), consistent with a close proximity of C/EBPβ and SUMO-1 since FRET can only occur between a donor and acceptor pair at distances less than 10 nm.

## Discussion

In this study we demonstrate that SUMO-1 and the enzymes Ubc9 and SENP1 of the SUMOylation process are expressed in primary astrocytes and that their expression is modified under inflammatory conditions. We also show that over-expressing these proteins modifies the astrocyte response to LPS, demonstrating a functional role for SUMOylation in astrocytes. To our knowledge this is the first report to characterize SUMO-1 and SRGs in astrocytes, confirming the existence and functionality of this important signaling pathway in one of the most abundant cell types in brain.

Our data shows that pro-inflammatory conditions that are induced by LPS or during normal aging, and as evidenced by a significant increase in the expression of NOS2 as well as C/EBPβ, decrease the expression levels of SUMO-1, Ubc9 and the protease SENP1, suggesting an anti-inflammatory role for SUMO-1 in brain. Although a decrease in SENP1 expression might be expected to promote inflammation (by reducing overall SUMOylation status), this enzyme is also involved in SUMO-1 maturation [41]. The fact that NA, an anti-inflammatory neurotransmitter, attenuates the effects of LPS on both the SRGs and NOS2 further supports an anti-inflammatory role of SUMO-1, and suggests that SUMOylation contributes to NA effects. Reduction in NA levels that occur during aging [40] could therefore contribute to the age-dependent decrease in SRGs.

In our experiments NOS2 promoter activity was decreased when SUMO-1, Ubc9, or SENP1 were over-expressed (Figure 3), suggesting an inhibitory role in primary astrocytes. SUMO-1 over-expression also decreased the activity of a pNFκB reporter plasmid that can only bind NFκB, which itself is not a SUMO target, therefore implying modulation of NFκB activity through interaction with other factors such as NEMO, IKK, or the inhibitory IκBα protein. Since NA increases both SRGs as well as IκBα [25], this raises the possibility that SUMOylation of IκBα contributes to the inhibitory effects of NA. However, the fact that over-expression of Ubc9 or SENP1 did not affect pNFκB activity suggests that their ability to inhibit NOS2 involves SUMOylation of proteins other than NFκB-related molecules.

The increase we observed in C/EBPβ mRNA levels caused by inflammatory stimuli are in accordance with previous studies [42-44]. Interestingly, and as we have shown before for the activation of NFκB [25], NA did not affect C/EBPβ mRNA levels although it reduced NOS2 mRNA. Several reports have shown that C/EBPβ activity is controlled by modulating its binding to DNA [45] and in many cell types NFκB and C/EBPβ co-operate in regulating NOS2 transcription [46,47]. This suggests that NA could inhibit NOS2 activation by altering the interaction of C/EBPβ with DNA and/or NFκB.

Although some reports suggest that other C/EBPs can be involved in the regulation of NOS2 expression [17,48] in this paper we have focused attention on the C/EBPβ isoform since in most studies NOS2 transcription has been shown to involve C/EBPβ. In rat C6 glioma cells, NOS2 induction by over-expression of MAPKs was blocked by a dominant negative form of C/EBPβ [49]. In human astrocytes, activation by the HIV-1 tat protein induced C/EBPβ expression and a dominant negative C/EBPβ blocked NO production [50]. In smooth muscle cells induction of NOS2 was accompanied by binding of C/EBPβ to the promoter [51], and in kidney cells, supershift assays revealed only the presence of C/EBPβ on the NOS2 promoter, when induced by LPS and IFNγ [52]. In liver cells, overexpression of the C/EBPβ-20 inhibitor LIP suppressed activity of the human NOS2 promoter [18]. Other inhibitors have also been shown to reduce NOS2 expression associated with a decrease in C/EBPβ levels or binding to the promoter [15,53,54].

Although several roles for SUMOylation in brain have been reported, there are limited studies describing the ability of SUMOylation to regulate brain inflammation. SUMOylation can have pro- or anti-inflammatory actions, depending on the specific SUMO isoform that is conjugated and the target protein. SUMO-1 levels were shown to be low in Parkinson's disease and Alzheimer's disease (AD) samples [55]; while SUMO-2/3 was detected at high levels in cortex after cerebral ischemia [38]. Transfection studies have shown that SUMOylation of amyloid precursor protein reduced Aβ production in human neurons [56] and in HeLA cells [57]; and that SUMO-1 modification increases human superoxide-dismutase-1 stability and aggregation [58]. The protein IRAK1 (interleukin-1 receptor associated kinase type 1) which transduces IL1 signaling and immune responses of Toll-like receptors is SUMOylated in brain, which could influence its ability to modulate inflammation [59].

SUMOylation is known to modulate transcription factor: DNA interactions [60,61] and in many cases has been shown to attenuate inflammatory responses [35,36,62]. SUMOylation of JunB modifies its ability to induce cytokine expression [63], SUMOylation of PPARγ increases it's interactions with the NCoR co-repressor and prevents inflammatory responses [37], SUMO-1 can modify several members of the NFκB signaling pathway, including NEMO (NFκB essential modulator) necessary for NFκB activation [64], as well as the inhibitory IκBα protein [34,65] and its kinase IKK [66]; and SUMOylation of STAT1 reduces IFN-dependent, STAT1 mediated transcription [67]. SUMOylation of C/EBPs has been shown to modulate transcription [26-29,68,69], and in many cases associated with reduced activity. An increase in SUMOylated C/EBPβ was shown to be involved in reduction of COX2 expression [70], and SUMOylation of C/EBPβ reduces albumin transcription [68]. Similarly, the synergic control elements of C/EBPα are believed to require SUMOylation for activity [29]. We therefore we considered the possibility that NA altered CEBP/β activity through SUMOylation.

There are several binding sites for members of C/EBP family on the rat NOS2 promoter, two of which are located close to the proximal NFκB binding site (C/EBP-2, located upstream; C/EBP-3, located downstream). ChIP results show that LPS treatment is associated with decreased levels of SUMO-1 associated with C/EBP-3; and that NA restores those levels (or prevents the LPS-induced loss). This suggests that SUMO-1 at this site exerts suppressive effects. We observed that C/EBPβ was bound to both site-2 and site-3, and was not significantly modified by LPS; however in the presence of NA there was a clear increase in C/EBPβ at site-2. Although C/EBPβ is generally considered to be pro-inflammatory, as indicated above there are also studies showing that it can suppress transcription. C/EBPβ represses activation of the cyclin D promoter [26]; and activation of c-Myc expression in Tcells [27].

Sequential immunoprecipitation experiments showing both C/EBPβ and SUMO-1 interacting at the C/EBP-3 site suggest that the SUMOylated factor at this site may be C/EBPβ. The results of Western blots and FRET analysis indicate strongly that C/EBPβ is SUMOylated however these results may also indicate the presence of a complex containing both C/EBPβ and SUMOylated factor(s). Thus, we conclude that the suppressive effects of NA on NOS2 expression involve increased SUMOylation at the NOS2 promoter.

Since in this paper we have focused attention on modification of C/EBPβ by SUMO-1, our results do not address the possibility that inflammatory stimuli or NA modify SUMO-2 or SUMO-3 interactions with the NOS2 promoter or with C/EBPβ, as recently reported to occur in COS1 cells [26]. Furthermore, in these studies we used an antibody directed against the amino terminus of C/EBPβ which does not distinguish between C/EBPβ isoforms; however findings that C/EBPβ1 is a preferential target for SUMOylation [26] suggests that our results may primarily reflect SUMOylation of this isoform.

## Conclusion

Our results demonstrate an important role for SUMOylation in regulating NOS2 expression in astrocytes, and point to modification of C/EBPβ as a critical determinant. However, there are likely to be other SUMO targets involved in the regulation of NOS2 transcription which may also contribute to suppressive effects of NA which need to be characterized. In view of the known involvement of NOS2 in a variety of neurological diseases and conditions, the knowledge that increasing SUMOylation processes can reduce NOS2 expression provides novel targets for therapeutic interventions.

## List of abbreviations

EAE: experimental acute encephalomyelitis; NFκB: nuclear factor κB; C/EBP: CCAAT enhancer-binding protein; MAPK: mitogen activated protein kinase; PIAS: protein inhibitors of activated stat; CRE: cAMP response element; CFP: cyan fluorescent protein; YFP: yellow fluorescent protein; CREB: cAMP response element binding protein; IL1: interleukin1; PPARγ: peroxisome-proliferator-activated-receptorγ; NCoR: nuclear receptor co-repressor; IκB: inhibitor of nuclear factor κB; IKK: IκB kinase; STAT: signal transducers and activators of transcription; QPCR: quantitative polymerase chain reaction; GDH: glyceraldehyde dehydrogenase.

## Competing interests

The authors declare that they have no competing interests.

## Authors' contributions

CAA designed and performed the experiments, analyzed the data, prepared figures and wrote the manuscript. DLF conceived and designed the study and contributed to the preparation of the manuscript.
